# Effects of two forms of school-based high-intensity interval training on body fat, blood pressure, and cardiorespiratory fitness in adolescents: randomized control trial with eight-week follow-up—the PEER-HEART study

**DOI:** 10.3389/fphys.2025.1530195

**Published:** 2025-04-08

**Authors:** Jarosław Domaradzki, Marek Popowczak, Katarzyna Kochan-Jacheć, Paweł Szkudlarek, Eugenia Murawska-Ciałowicz, Dawid Koźlenia

**Affiliations:** Faculty of Physical Education and Sport, Wroclaw University of Health and Sport Sciences, Wroclaw, Poland

**Keywords:** physical education, adolescent, body composition, cardiovascular fitness, health, school-based setting, plyometric exercises

## Abstract

**Introduction:**

This study examined the effects of 8-week interventions based on two variants of typical exercises, namely, high-intensity interval training (HIIT) and high-intensity plyometric training (HIPT), on body fat (BF%), blood pressure, and cardiorespiratory fitness (CRF). In addition, the sustainability of the effects after another 8 weeks was assessed.

**Methods:**

The project was designed as a randomized controlled trial with eight groups of participants (two variants, two sexes, and two groups (experimental and control)) and was conducted in a school physical education (PE) program. The outcomes analyzed were the BF%, systolic (SBP), diastolic blood pressure (DBP), and CRF expressed in terms of maximum oxygen uptake (VO_2max_). A total of 307 healthy adolescents participated in this study and were randomly assigned into the two groups. During the 8 weeks, the participants completed two exercise sessions each week with progressively increasing volumes. For the first 2 weeks, the sessions involved four rounds of 20 s of intense effort followed by 10 s of rest; this increased to six rounds during weeks 3–4 and eight rounds during weeks 5–8. The HIPT program was based on plyometric exercises, whereas the HIIT was based on bodyweight resistance exercises.

**Results:**

Multidimensional analysis of variance (ANOVA) indicated a statistically significant second-order interaction (time × variant × group: Ʌ = 0.943, *F* = 2.20, *p* < 0.027, η^2^_pG_ = 0.057, d = 0.25), confirming the changes in the BF%, SBP, DBP, and VO_2max_ dependent on the type of intervention and group assignment. The ANOVA results revealed significant main and interaction effects for BF%, SBP, and DBP, with time and the HIIT variant as the main contributors (BF%: *F* = 3.911, *p* = 0.023, η^2^_pG_ = 0.001, d = 0.04 vs. *F* = 9.900, *p* < 0.001, η^2^_pG_ = 0.001, d = 0.03; SBP: *F* = 31.801, *p* < 0.001, η^2^_pG_ = 0.012, d = 0.16 vs. *F* = 8.939, *p* = 0.003, η ^2^_pG_ = 0.026, d = 0.16; DBP: *F* = 3.470, *p* = 0.033, η^2^_pG_ = 0.002, d = 0.06 vs. *F* = 4.982, *p* = 0.026, η^2^_pG_ = 0.014, d = 0.12). The second-order interaction for VO_2max_ (time × sex × group: *F* = 6.960, *p* = 0.001, η^2^_pG_ = 0.003, d = 0.05) indicated that the improvements over time were not related to the training variant. Although these effects were small (low eta values), post hoc tests (all comparisons in post-intervention, *p* > 0.05) showed that both the HIIT and HIPT groups exhibited beneficial changes compared to controls; however, no statistically significant differences were observed between the experimental and control groups. Furthermore, the observed improvements were maintained through the 8-week follow-up period, as demonstrated by no significant changes between the post-intervention and follow-up measurements (*p* > 0.05). Discriminant analysis showed that BF% and SBP were the key variables for the two exercise variants in men, with HIPT yielding greater reductions in SBP and HIIT resulting in more pronounced decreases in BF%.

**Discussion:**

In conclusion, both HIIT and HIPT interventions effectively improved health-related parameters, providing valuable enrichment to the PE lessons in schools. These benefits were also sustained for at least 8 weeks post-intervention.

## 1 Introduction

Over the past three decades, obesity among youth has become a global health concern because of its increasing prevalence. According to data from the World Health Organization, over 390 million children and adolescents aged 5–19 years were overweight in 2022, including 160 million affected by obesity (World Health Organization, 2023). The projections indicate that the obesity rates could reach 20% in boys and 18% in girls by 2035 (Lobstein et al., 2023). This situation is particularly concerning in Poland, where approximately 29% of the adults were obese in 2019 (Pawlewicz, 2024); forecasts predict further growth of this number, with obesity affecting more than 35% of men and 25% of women by 2035 (Lobstein et al., 2023). Furthermore, the WHO European Region has yet to implement measures to tackle this epidemic (World Health Organization Regional Office for Europe, 2021). The health consequences of obesity in youth are severe and tend to worsen in adulthood. Obese adolescents are prone to higher risks of cardiometabolic diseases, including hypertension, type 2 diabetes, and atherosclerosis, which could lead to increased morbidity and mortality compared to their normal-weight peers (Després et al., 2008; Kumar and Kelly, 2017; Di Angelantonio et al., 2016; Baker et al., 2007; Weihrauch-Blüher et al., 2019). In addition, obesity negatively impacts mental health, contributing to depression, reduced quality of life, discrimination, and poor academic performance (Pont et al., 2017; Ma et al., 2021; Ladd et al., 2017). A study across 32 countries found that normal-weight children have a 13% higher chance of better academic performance than those with obesity (Devaux and Vuik, 2019).

Regular physical activity is a key intervention for preventing obesity and improving metabolic health in youth. High-intensity interval training (HIIT) has been shown to be effective in improving body composition, increasing cardiorespiratory fitness (CRF), and reducing cardiovascular risk (Carson et al., 2014; Khalafi et al., 2022; Robinson et al., 2015). Owing to its short duration and efficiency, HIIT is particularly appealing in school settings, where it offers significant health benefits with less time commitment from youth who may otherwise be discouraged by longer activities (Weston et al., 2021; Chuensiri et al., 2018). Physical education (PE) classes typically lasting 45 min can provide an ideal environment for integrating HIIT, especially when using the Tabata protocol (Peake et al., 2015). Recent studies have shown that prolonged exercise sessions during PE in schools are unattractive to youth, resulting in decreased motivation and lower exercise intensity (Weston et al., 2021; Duncombe et al., 2024). Thus, identifying student-friendly exercise formats that can sustain high intensity is essential. Comparative experiments have demonstrated varying effectiveness among exercise variants (Murawska-Ciałowicz et al., 2021). Our previous research that used a modified Tabata program was shown to reduce body fat, lower blood pressure, and improve CRF; however, the intensity and motivation declined over time (Domaradzki et al., 2020, 2022). In the present study, we introduce high-intensity plyometric training (HIPT) as a potentially more engaging and effective variant for students; its effectiveness has been supported by studies in athletes (Váczi et al., 2013; Söyler et al., 2024). The protocol emphasizes plyometric exercises aimed at maximizing rapid repetitions and total work done (Davies et al., 2015). Such explosive movements engage the fast-twitch muscle fibers, potentially enhancing muscle power and anaerobic performance. Plyometric training is characterized by rapid eccentric-concentric transitions that can improve motor function, muscle strength, and endurance in youth (Staniszewski et al., 2024). Combined with HIIT, it may further enhance muscle strength, power, and local muscular endurance (Hammami et al., 2021; Moran et al., 2017). For instance, Racil et al. (2016) found that adding plyometric exercises to a HIIT program improved body composition and jumping ability in obese adolescent girls. For comparison, we introduce two variants in the present study: the standard HIIT protocol (Domaradzki et al., 2020, 2022) and a modified HIPT protocol incorporating plyometric exercises. Both programs were administered with the aim of maximizing the number of repetitions during the work phase, thereby increasing the demands on the cardiovascular–respiratory systems and energy metabolism. In our present study, the HIIT program entailed bodyweight resistance exercises to impose a greater load on the musculoskeletal system, whereas the HIPT program utilized high-speed plyometric exercises to facilitate a higher number of repetitions, potentially increasing the total work performed. Further research on HIIT variants, including those with plyometric elements, are crucial for developing effective school health programs, especially considering that the sustainability of these effects remain unexplored in school settings.

Therefore, the aim of the present study was twofold: (1) to determine whether body fat percentage (BF%), systolic and diastolic blood pressure (SBP/DBP), and CRF change after 9 weeks of high-intensity training using the two intervention variants, namely, HIIT and HIPT; (2) to assess the sustainability of the results induced by these interventions 8 weeks after completion. Many studies lack assessments of the durability effects and focus only on the intervention period. It is hypothesized that integrating both types of interventions into PE classes could serve as time-efficient and effective methods for enhancing physical fitness while mitigating obesity-related health issues among youth. Specifically, both programs are expected to significantly reduce body fat percentage and improve CRF and blood pressure compared to the baseline values. Moreover, we anticipate that the HIPT variant will produce greater improvements in these parameters by allowing a higher number of repetitions through high-speed plyometric movements. Finally, these beneficial effects are expected to be sustained for at least 8 weeks after completion of the intervention.

## 2 Materials and methods

### 2.1 Ethics committee

The Ethics Committee of the Wroclaw University of Health and Sport Sciences granted approval for this study (ECUPE no. 33/2018; approval date: 31 October 2018). The research adhered to all ethical principles for medical research involving human subjects, as outlined in the Declaration of Helsinki by the World Medical Association.

### 2.2 Clinical trial registration

The study was conducted in Wroclaw, Poland, in 2024 as project supported financially by the state budget under the Polish Ministry of Science and Higher Education program titled “Science for Society II” (project no. NdS-II/SP/0521/2023/01). The study has been registered as a clinical trial at clinicaltrials.gov with ID: NCT06431230 and the acronym PEER-HEART for “Physical Education dosE Response Health markErs Adolescents inteRval Training.”

### 2.3 Participants

**FIGURE 1:**
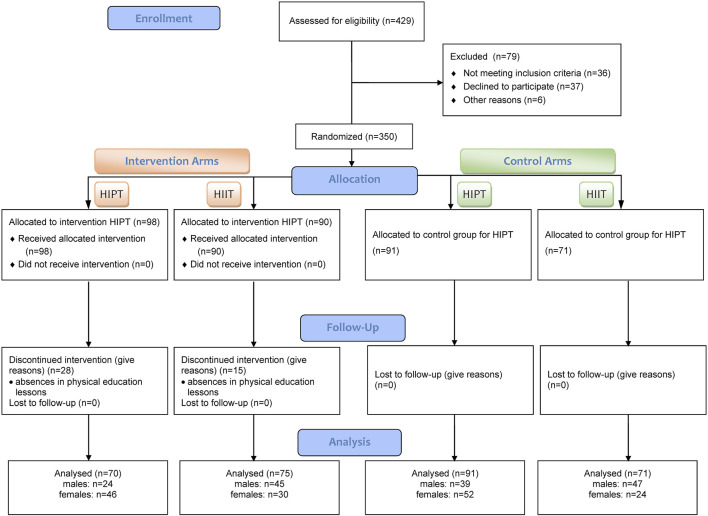
Flowchart depicting the process of participant inquiry.

G*Power v.3.1 (Faul et al., 2007) software was used for the basic calculations by assuming the multidimensional analysis of variance (MANOVA) method for eight groups (two schools, boys and girls separately under the experimental and control groups) and three repeated measurements (baseline, post-intervention, and follow-up). For an effect size of 0.20, an alpha level of 0.05, and a power of 0.95, the minimum sample size required was 310 participants. The study sample consisted of individuals from two schools, each participating in two versions of high-intensity training, namely, the plyometry-based HIPT method and the previously used modified Tabata protocol (HIIT) as the standard, along with two control groups from both schools. Initially, 429 participants were assigned to the project across thirteen classes in each school. The group assignments were determined through a simple, non-returnable randomization using the online tool available at www.randomization.com (accessed on 08.01.2024). Each class was labeled with a number from 1 to 13 in both schools, and the students from each class were at the same educational level and followed the same PE curriculum. The HIIT inclusion criteria were as follows: the participants must be first-year high school students who are regularly enrolled in and participate in PE classes; they must be free from any medical conditions or contraindications, such as cardiovascular, respiratory, or musculoskeletal disorders, that could prevent them from safely engaging in high-intensity exercises; they should not be involved in any additional structured high-intensity training programs outside the standard school curriculum; lastly, informed consent must be provided, and parental or guardian consent must be obtained for minors. Prior to the group allocations, some of the subjects were excluded owing to refusal to participate, medical contraindications, or participation in additional sports activities within the past 6 months (n = 79). A few more students were excluded during the intervention due to absence exceeding 20% during the PE lessons (n = 43). No adverse effects were observed from the HIIT interventions. The final analysis included (n = 307) adolescent students. The flow structure of participant selection for the interventions is detailed in Figure 1. Participation was voluntary, and the students could withdraw at any time. Both students and their parents/guardians were informed about the study objectives and procedures. Written informed consent was obtained from the school principals, parents, and study subjects before participation.

### 2.4 Anthropometric measurements

#### 2.4.1 Procedures

Morphological measurements were obtained at three time points: before the 8-week intervention, immediately after the intervention, and at the end of an 8-week follow-up period. These assessments were conducted on a single day between 8:00 a.m. and 1:00 p.m. in sports halls under standardized conditions for each group. The participants wore T-shirts, shorts, and shoes for the tests, although the anthropometric measurements were obtained without shoes. First, the body height and composition were measured. On a different day, the blood pressure was measured, following which the beep test was performed. The students, parents, and teachers were informed of the specific rules for all tests before commencement of the project. All procedures were conducted thrice during the three time points mentioned above.

#### 2.4.2 Body morphology

Body height measurements were obtained to the nearest 0.1 cm using anthropometers (GPM Anthropological Instruments, DKSH Ltd., Switzerland). The body height was additionally measured using an anthropometer in the standing position barefoot according to the International Society for the Advancement of Kinanthropometry (ISAAK) (Marfell-Jones et al., 2006). The bodyweight and BF% were measured with a Tanita Inner Scan V (BC-601 model, Tanita Co., Tokyo, Japan). The reliabilities of these methods were verified in a prior research by Ramírez-Vélez et al. (2016). Prior to the measurements, the students received instructions on the examination procedures. They were asked to void their bladders, avoid excessive water intake, and maintain their usual breakfast routines. For the measurements, the participants were required to be barefoot and shirtless, ensuring that their heels were placed on the rear electrodes of the scale, with legs straight at the knee and hip joints, arms slightly abducted and flexed at the shoulder joints, elbows straight, and fingers touching the manual electrodes. These measurements were conducted in the evening at least 3 h after the last meal of the day. The body mass index (BMI) was calculated as weight in kilograms divided by height in meters squared (kg/m^2^).

### 2.5 Blood pressure measurements

All blood pressure measurements were performed using an Omron BP710 Automatic Blood Pressure Monitor (Omron Healthcare, Inc., Hoffman Estates, IL, United States) (Nasir et al., 2021). The appropriate cuff sizes were selected based on the participants’ upper arm circumferences. The participants were asked to sit quietly for 10 min before the measurements, and the blood pressure readings were acquired three times at 10-min intervals. The average of these three readings was used for the analysis. The blood pressure readings were measured before and after the intervention period as well as during follow-up.

### 2.6 Multistage fitness test

To assess the maximal heart rate, we performed the multistage fitness test (MSFT), and the participants were measured using the Polar Verity Sense sensors (Polar Electro, Kempele, Finland) (Navalta et al., 2023). The MSFT involves continuous running between two lines spaced 20 m apart and synchronized to a series of recorded beeps. The participant starts running at a speed of 8.5 km/h, stops at each marked line, turns 180°, and runs back. As the test progresses, the pace increases by 0.5 km/h every minute, which is indicated by a sound signal. The test continues until the participant can no longer maintain the required speed. The maximum oxygen uptake (VO_2max_) was calculated based on the following formula (Ramsbottom et al., 1988):

$$\text{VO}2\max=3.46\;*\;\left(L+\text{SN}/\left(L\;*\;0.4325+7.0048\right)\right)+12.2,$$

where L is the level and SN is the number of shuttles.

### 2.7 Interventions

The HIIT program based on the Tabata method was implemented during two PE classes each week over an 8-week period, where the training volume increased progressively. The intensity of the workouts was monitored using the Polar Verity Sense devices (Polar Electro, Kempele, Finland), and the target intensity was set to a specific percentage of the maximum heart rate. In the other PE classes, the students followed the school’s standard curriculum for first-year students, which focused on improving their skills in various sports. The Polar Verity Sense sensors were used for monitoring the heart rate to control the effort intensity target at 75%HR_max_ as established by the beep test (Duncombe et al., 2024).

**FIGURE 2:**
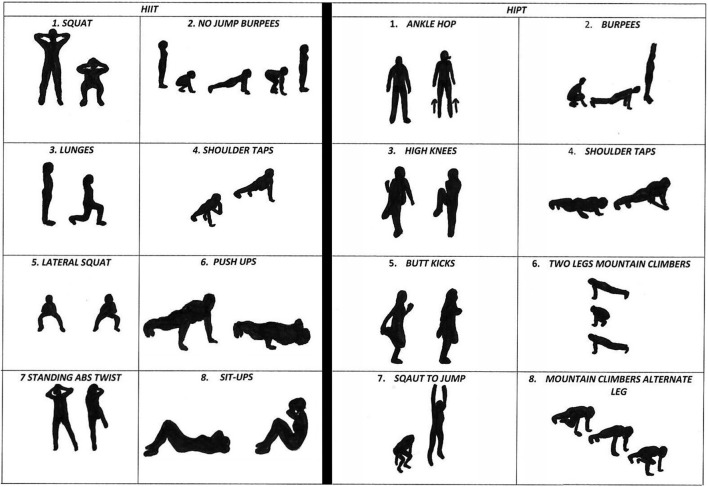
Intervention structures for the two training variants.

During the sessions incorporating the intervention protocol, the students began with a 10-min standardized warm-up. Then, they engaged in HIIT exercises comprising four rounds of 20 s of intense effort, followed by 10 s of rest for the first 2 weeks; this pattern shifted to six rounds during the third and fourth weeks and increased to eight rounds during the final 4 weeks (Table 1). The HIPT program included exercises such as ankle hops, burpees, high knees, shoulder taps to hand claps, butt kicks, two-leg mountain climbers, squat jumps, and alternating leg mountain climbers. The HIIT program featured squats, no-jump burpees, lunges, shoulder taps, lateral squats, push-ups, standing abs twists, and sit-ups. All sessions of the intervention program were supervised. Students received constant feedback over the duration of each work and rest rounds from the supervisor, and they could also see the timer. All exercise rounds were performed in an “all-out” manner. The students were constantly motivated to perform as many repetitions as possible. Subsequently, a standard PE program was implemented to develop comprehensive motor skills, including both team games (e.g., soccer, volleyball, and basketball) and individual disciplines (e.g., athletics and gymnastics). The lesson structure for the control groups was identical, except for the absence of the HIIT and HIPT interventions. The two intervention structures are presented in Figure 2.

**TABLE 1:** Weekly HIIT variant intensities vs. percentage of the maximal heart rate [%].

Week	HIPT (n = 70)		HIIT (n = 75)	
	Mean ± SD (95% CI) [%]	Min–max [%]	Mean ± SD (95% CI) [%]	Min–max [%]
1	80.7 ± 6.8 (79.1–82.3)	65–97	85.7 ± 6.1 (84.3–87.1)	66–97
2	79.7 ± 6.8 (78.1–81.3)	61.9–94.8	82.4 ± 5.8 (81–83.7)	66.1–96.2
3	78.4 ± 8.2 (76.4–80.3)	52.6–90.1	81.3 ± 6.2 (79.9–82.7)	65.3–92.5
4	78.7 ± 7.7 (76.9–80.6)	53.1–92	81.2 ± 6.3 (79.8–82.7)	62.7–93.8
5	76.4 ± 8.6 (74.3–78.4)	48.7–90.4	79.2 ± 8 (77.4–81.1)	52.9–93
6	76 ± 9.5 (73.7–78.3)	49.1–89.9	78.5 ± 8 (76.6–80.3)	41.3–91.7
7	77.4 ± 6.6 (75.8–79)	62.4–91.8	79.5 ± 6.3 (78.1–81)	57.9–90.9
8	75.9 ± 8.6 (73.8–77.9)	56.5–90.6	80.9 ± 8.5 (79–82.9)	50.7–96.5

The mean resting heart rate (HR) values for the four groups of men (HIIT E, HIIT C, HIPT E, and HIPT C) were assessed at three time points: before intervention (Pre), immediately after intervention (Post), and at follow-up (FU). In the HIIT E group, the measured HRs were 72.3 ± 7.5 bpm (70.1–74.0) at Pre, 72.1 ± 6.3 bpm (70.2–74.0) at Post, and 76.0 ± 8.9 bpm (73.3–78.7) at FU; similarly, the values for the HIIT C group were 71.5 ± 6.3 bpm (69.7–73.4) at Pre, 72.6 ± 6.9 bpm (70.5–74.6) at Post, and 72.2 ± 8.5 bpm (69.5–74.5) at FU. In the HIPT E group, the measured HRs were 75.0 ± 5.7 bpm (72.6–77.5) at Pre, 75.6 ± 5.6 bpm (73.3–78.0) at Post, and 71.5 ± 7.1 bpm (68.5–74.6) at FU; similarly, the values for the HIPT C group were 73.4 ± 6.8 bpm (71.2–75.6) at Pre, 71.3 ± 6.4 bpm (69.2–73.4) at Post, and 71.0 ± 8.5 bpm (68.5–73.7) at FU. The mean resting HR values for the four groups of women were as follows: in the HIIT E group, the measured HRs were 72.1 ± 5.7 bpm (69.9–74.3) at Pre, 74.9 ± 6.3 bpm (72.6–77.1) at Post, and 72.0 ± 8.3 bpm (68.9–75.2) at FU; in the HIIT C group, the measured HRs were 72.8 ± 8.5 bpm (69.2–76.4) at Pre, 70.8 ± 7.4 bpm (67.7–73.9) at Post, and 76.2 ± 7.4 bpm (73.1–79.3) at FU; in the HIPT E group, the measured HRs were 75.0 ± 6.0 bpm (73.2–76.8) at Pre, 74.7 ± 6.1 bpm (72.9–76.9) at Post, and 74.3 ± 8.6 bpm (71.7–76.9) at FU; in the HIPT C group, the measured HRs were 74.1 ± 5.8 bpm (72.4–75.7) at Pre, 74.3 ± 6.7 bpm (72.4–76.1) at Post, and 74.2 ± 8.7 bpm (71.8–76.7) at FU. All data values are presented as mean ± standard deviation, with the 95% confidence intervals shown within parentheses.

### 2.8 Statistics

Before conducting the statistical analysis, all assumptions were verified and the usefulness of the chosen methods was confirmed. Given the large sample size, the Kolmogorov–Smirnov test was used to assess the normality of the continuous variable distributions. A slight deviation from normal distribution was observed for body fat values; in this case, the inference was confirmed using non-parametric tests, including the Kruskal–Wallis analysis of variance (ANOVA) for comparisons across the groups or the Wilcoxon signed-rank test for repeated measures within each group. When the interpretation of results from both methods was consistent, it was assumed that the parametric ANOVA was robust against violations of variance homogeneity assumptions. MANOVA and mixed-effect analysis of variance (ME ANOVA) were employed to test the effects of the interventions on the studied variables. Four-way models were used with sex (males (M) and females (F) as levels), group (experimental (E) and control (C) as levels), intervention variants (levels) as the between-group factors, and time (three points: pre-intervention, post-intervention, and follow-up denoted by PRE, POST, and FU, respectively, as levels) as the within-group factors. Before analysis, the homoscedasticity and sphericity were tested using Levene’s and Mauchly’s tests, respectively, along with the Greenhouse–Geisser correction when the sphericity assumption was violated. The effect size was assessed using the generalized partial eta square (η^2^_pG_) and converted to Cohen’s d value (d) (Lakens, 2013). In cases where we observed significant effects, a post hoc test with Bonferroni correction was used. All procedures were conducted twice, once for the basic anthropometric measurements to study the general differences between the groups and then for the outcomes of interest, namely, BF%, SBP, DBP, and CRF (in terms of VO_2max_). The effects between the two variants were compared using the discriminant analysis (DA) method; this approach allows identification of the set of variables that can best distinguish between the two categories, which unlike the ANOVA enables multivariate analyses. Standard DA was conducted using the delta (Δ) of the body fat percentages, blood pressure values, and maximum oxygen uptake as pre–post differences, with Wilks’ Lambda value set as the criterion for assessment of the model built using all four variables. In addition, partial Wilks’ Lambda value was calculated as the individual load of each variable on the model. Moreover, the tolerance values were calculated as (1-R^2^) for each variable in relation to the remaining variables. Tolerance is defined as the proportion of a variable’s variance that is not accounted for by other independent variables in the equation. The alpha level was fixed at α = 0.05, and calculations with *p* < 0.05 were considered to be statistically significant. All analyses were conducted using Statistica 13.0 software (StatSoft Poland 2018, Cracow, Poland) and R software with RStudio (PBC, Boston, MA, United States; http://www.rstudio.com/) (accessed on 15 September 2024).

## 3 Results

### 3.1 General sample characteristics: basic anthropometric measurements

Descriptive statistics of the baseline, post-intervention, and follow-up anthropometric measurements are presented in Table 2. MANOVA was conducted for the basic anthropometric measurements and showed statistically significant multidimensional main effects for all four factors: variant (Ʌ = 0.941, *F* = 6.13, *p* < 0.001, η^2^_pG_ = 0.058, d = 0.35), sex (Ʌ = 0.716, *F* = 39.20, *p* < 0.001, η^2^_pG_ = 0.284, d = 0.89), group (Ʌ = 0.936, *F* = 6.74, *p* < 0.001, η^2^_pG_ = 0.064, d = 0.37), and time (Ʌ = 0.002, *F* = 22,430.06, *p* < 0.001, η^2^_pG_ = 0.998, d = 22.3). There were no statistically significant interactions between the factors, except for time: time × variant (Ʌ = 0.902, *F* = 5.30, *p* < 0.001, η^2^_pG_ = 0.098, d = 0.33), time × sex (Ʌ = 0.523, *F* = 44.74, *p* < 0.001, η^2^_pG_ = 0.477, d = 0.95), and time × group (Ʌ = 0.909, *F* = 4.89, *p* < 0.001, η^2^_pG_ = 0.091, d = 0.32). This confirmed that the interventions affected the basic anthropometric features; however, the results were dependent on the type of HIIT and were related to sex. ANOVA showed changes over time related to the natural growth of the adolescents in terms of height (*F* = 601.832, *p* < 0.001, η^2^_pG_ = 0.002, d = 0.05) and sexual dimorphism in height (*F* = 265.809, *p* < 0.001, η^2^_pG_ = 0.470, d = 1.33). There were significant sexual differences in bodyweight (*F* = 54.570, *p* < 0.001, η^2^_pG_ = 0.019, d = 0.20), but the effects were related to the variant of HIIT used (*F* = 5.725, *p* < 0.002, η^2^_pG_ = 0.019, d = 0.20). Similarly, the effects on BMI mirrored differences in bodyweights (sex: *F* = 54.571, *p* < 0.001, η^2^_pG_ = 0.018, d = 0.19; variant: *F* = 5.720, *p* < 0.002, η^2^_pG_ = 0.018, d = 0.19). Detailed comparisons with the post hoc tests revealed no significant differences in any of the variables (all *p* > 0.1) at baseline.

**TABLE 2:** Descriptive statistics of the basic anthropometric measurements of the pre-intervention (Pre), post-intervention (Post), and follow-up (FU) values based on variant, sex, and group.

Variable	Time	HIIT		HIPT	
		Men			
		E (n = 45)	C (n = 47)	E (n = 24)	C (n = 39)
		Mean ± SD (95% CI)	Mean ± SD (95% CI)	Mean ± SD (95% CI)	Mean ± SD (95% CI)
Age [years]	Pre	15 ± 0.6 (14.8–15.3)	15.1 ± 0.6 (14.9–15.2)	14.9 ± 0.5 (14.8–15.1)	15 ± 0.4 (14.9–15.1)
Body height [cm]	Pre	175.4 ± 6.6 (172.6–178.2)	178.1 ± 6.8 (175.9–180.4)	176 ± 6.1 (174.2–177.9)	176.7 ± 6.9 (174.7–178.8)
	Post	175.7 ± 6.6 (172.9–178.4)	178.5 ± 6.8 (176.3–180.7)	176.3 ± 6.1 (174.5–178.2)	177.1 ± 7 (175–179.1)
	FU	176 ± 6.6 (173.2–178.8)	178.8 ± 6.8 (176.6–181)	176.8 ± 6.1 (174.9–178.6)	177.5 ± 7.1 (175.4–179.5)
Bodyweight [kg]	Pre	65.5 ± 9 (61.7–69.3)	68.8 ± 16. 6 (63.4–74.2)	65.2 ± 11.6 (61.7–68.7)	62.6 ± 8.7 (60–65.1)
	Post	64.9 ± 7.7 (61.6–68.1)	69.7 ± 16.8 (64.2–75.1)	64.5 ± 10.6 (61.4–67.7)	63.2 ± 9.5 (60.4–66)
	FU	64.8 ± 8.6 (61.1–68.4)	69.7 ± 16.6 (64.3–75.1)	65.5 ± 11.1 (62.1–68.8)	63.4 ± 8.5 (60.9–65.9)
Body mass index [kg/m^2^]	Pre	21.3 ± 2.8 (20.1–22.5)	21.5 ± 3.9 (20.2–22.7)	21 ± 3.3 (20–22)	20 ± 2.3 (19.3–20.7)
	Post	21 ± 2.5 (20–22.1)	21.7 ± 3.9 (20.4–22.9)	20.7 ± 3 (19.8–21.6)	20.1 ± 2.6 (19.4–20.9)
	FU	20.9 ± 2.7 (19.8–22)	21.6 ± 3.9 (20.3–22.9)	20.9 ± 3.2 (20–21.9)	20.1 ± 2.2 (19.5–20.7)

Women					
Variable	Time	E (n = 30)	C (n = 24)	E (n = 46)	C (n = 52)
Age [years]	Pre	15.1 ± 0.5 (14.9–15.2)	15 ± 0.6 (14.9–15.2)	14.8 ± 0.4 (14.6–14.9)	15 ± 0.4 (14.8–15.2)
Body height [cm]	Pre	165.6 ± 6 (163.9–167.4)	165.2 ± 6.2 (163.5–166.9)	162.9 ± 5.4 (160.8–164.9)	164 ± 5.7 (161.6–166.4)
	Post	165.8 ± 5.9 (164–167.6)	165.5 ± 6.2 (163.8–167.2)	163.2 ± 5.4 (161.2–165.2)	164.3 ± 5.7 (161.9–166.8)
	FU	166.1 ± 6 (164.3–167.9)	165.7 ± 6.2 (164–167.4)	163.6 ± 5.3 (161.6–165.5)	164.7 ± 5.8 (162.2–167.1)
Bodyweight [kg]	Pre	58.2 ± 10.9 (55–61.5)	57.3 ± 8.3 (54.9–59.6)	54.2 ± 6.5 (51.8–56.7)	55.8 ± 7.5 (52.7–59)
	Post	58.4 ± 9.9 (55.4–61.3)	58.1 ± 8.7 (55.7–60.5)	54 ± 5.9 (51.9–56.2)	56 ± 7.5 (52.8–59.1)
	FU	58.1 ± 10.4 (55–61.1)	57.5 ± 8.7 (55–59.9)	54.7 ± 6.6 (52.3–57.2)	56.2 ± 7.5 (53–59.4)
Body mass index [kg/m^2^]	Pre	21.2 ± 3.6 (20.1–22.3)	21 ± 3 (20.2–21.8)	20.5 ± 2.4 (19.6–21.4)	20.8 ± 2.8 (19.6–22)
	Post	21.2 ± 3.2 (20.2–22.2)	21.2 ± 3 (20.4–22.1)	20.3 ± 2.1 (19.5–21.1)	20.7 ± 2.6 (19.6–21.8)
	FU	21 ± 3.4 (20–22)	20.9 ± 3 (20.1–21.8)	20.5 ± 2.4 (19.6–21.4)	20.7 ± 2.6 (19.6–21.8)

### 3.2 Detailed effects on the outcomes of interest

**FIGURE 3:**
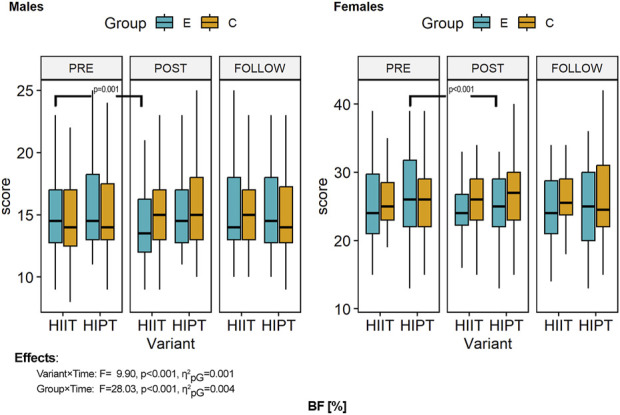
Within- and between-group differences to the HIIT interventions in body fat percentage. ANOVA, analysis of variance; PRE, preintervention; POST, postintervention; FU, follow-up; E, experimental group; C, control group; HIIT, high-intensity interval training (previously used variant); HIPT, high-intensity plyometric training (plyometric HIIT variant).

**FIGURE 4:**
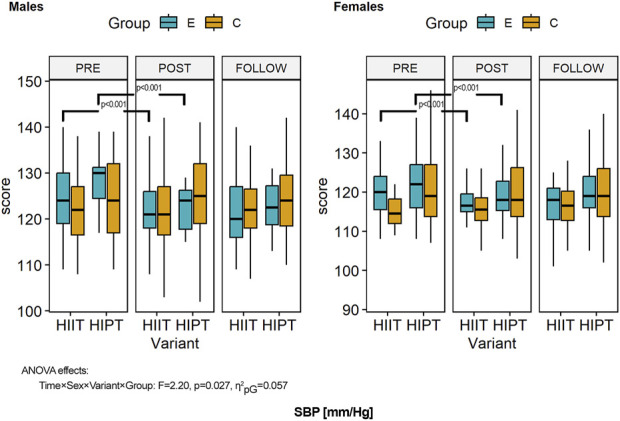
Within- and between-group differences to the HIIT interventions in systolic blood pressure (SBP). ANOVA, analysis of variance; PRE, preintervention; POST, postintervention; FU, follow-up; E, experimental group; C, control group; HIIT, high-intensity interval training (previously used variant); HIPT, high-intensity plyometric training (plyometric HIIT variant).

**FIGURE 5:**
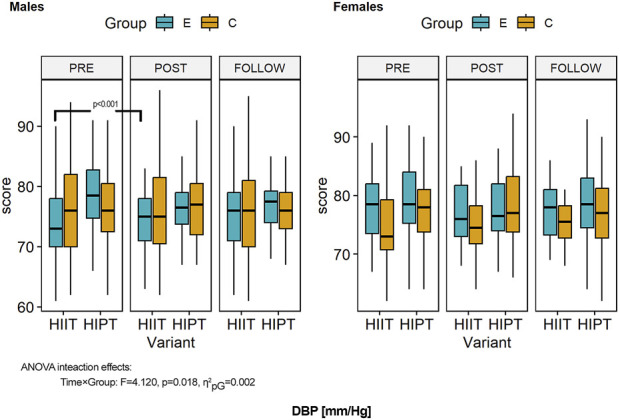
Within- and between-group differences to the HIIT interventions in diastolic blood pressure (DBP). ANOVA, analysis of variance; PRE, preintervention; POST, postintervention; FU, follow-up; E, experimental group; C, control group; HIIT, high-intensity interval training (previously used variant); HIPT, high-intensity plyometric training (plyometric HIIT variant).

**FIGURE 6:**
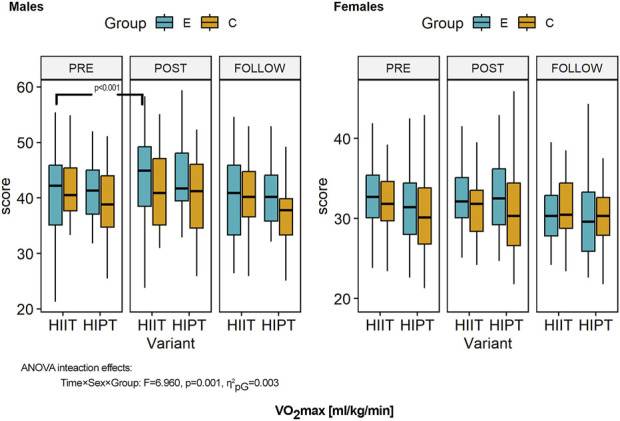
Within- and between-group differences to the HIIT interventions in cardiorespiratory fitness (measured in terms of VO_2max_). ANOVA, analysis of variance; PRE, preintervention; POST, postintervention; FU, follow-up; E, experimental group; C, control group; HIIT, high-intensity interval training (previously used variant); HIPT, high-intensity plyometric training (plyometric HIIT variant).

The descriptive statistics of the outcomes of interest at baseline, post-intervention, and follow-up are presented in Table 3 and illustrated in Figures 3–6. MANOVA showed statistically significant multidimensional main effects on all four factors: variant (Ʌ = 0.968, *F* = 2.41, *p* = 0.049, η^2^_pG_ = 0.032, d = 0.26), sex (Ʌ = 0.546, *F* = 61.43, *p* < 0.001, η^2^_pG_ = 0.454, d = 1.3), group (Ʌ = 0.942, *F* = 4.58, *p* < 0.001, η^2^_pG_ = 0.058, d = 0.35), and time (Ʌ = 0.006, *F* = 5,683.87, *p* < 0.001, η^2^_pG_ = 0.994, d = 12.8). The effect of the time factor, which was actually an intervention effect, was immense; however, it was related to the variant and group but not to sex. This was confirmed through the second-order interaction term (time × variant × group: Ʌ = 0.943, *F* = 2.20, *p* < 0.027, η^2^_pG_ = 0.057, d = 0.26).

**TABLE 3:** Descriptive statistics of the body fat, systolic and diastolic blood pressures (SBP/DBP), and VO_2max_ values at pre-intervention (Pre), post-intervention (Post), and follow-up (FU) based on variant, sex, and group.

Variable	Time	HIIT		HIPT	
		Men			
		E (n = 45)	C (n = 47)	E (n = 24)	C (n = 39)
		Mean ± SD (95% CI)	Mean ± SD (95% CI)	Mean ± SD (95% CI)	Mean ± SD (95% CI)
Fat [%]	Pre	15.8 ± 3.4 (14.4–17.2)	16.9 ± 5.6 (15–18.7)	16.4 ± 5.8 (14.7–18.2)	14.8 ± 3.5 (13.8–15.8)
	Post	15.1 ± 3 (13.8–16.4)	17.3 ± 5.8 (15.4–19.1)	15.1 ± 4.5 (13.8–16.5)	15.1 ± 3.3 (14.1–16)
	FU	15.1 ± 3.3 (13.7–16.5)	16.4 ± 5.8 (14.5–18.3)	16.1 ± 5.1 (14.6–17.6)	15.3 ± 2.9 (14.4–16.1)
SBP [mm/Hg]	Pre	127.8 ± 5.7 (125.4–130.2)	124.5 ± 8.6 (121.7–127.3)	124.6 ± 8.9 (121.9–127.3)	122.5 ± 7.4 (120.3–124.6)
	Post	122.5 ± 4.7 (120.5–124.4)	124.9 ± 9.4 (121.9–127.9)	121.8 ± 6.6 (119.9–123.8)	122.4 ± 8.5 (119.9–124.9)
	FU	122.7 ± 5.6 (120.3–125.1)	123.8 ± 7.9 (121.2–126.3)	121.4 ± 7.7 (119.1–123.7)	122.1 ± 6.6 (120.2–124.1)
DBP [mm/Hg]	Pre	78.8 ± 7.1 (75.7–81.8)	76.6 ± 7.3 (74.2–79)	74.6 ± 8.1 (72.2–77)	76.1 ± 7.9 (73.8–78.4)
	Post	76.1 ± 5.1 (74–78.3)	76.6 ± 5.7 (74.8–78.4)	74.4 ± 5.9 (72.6–76.2)	75.8 ± 8.1 (73.4–78.2)
	FU	76.6 ± 4.4 (74.7–78.4)	76.2 ± 5.3 (74.5–77.9)	75.5 ± 6.7 (73.5–77.5)	75.7 ± 8.1 (73.3–78.1)
VO_2max_ [mL/kg/min]	Pre	41.1 ± 5.8 (38.7–43.5)	39 ± 6.9 (36.8–41.3)	40.1 ± 8 (37.7–42.4)	42.3 ± 6.1 (40.5–44.1)
	Post	43.2 ± 6.4 (40.5–45.9)	40.5 ± 7.8 (37.9–43)	43.9 ± 8.5 (41.3–46.4)	41.6 ± 6.8 (39.6–43.6)
	FU	40.7 ± 6 (38.1–43.2)	37.5 ± 5.1 (35.8–39.17)	40.3 ± 7.7 (38–42.6)	40.6 ± 6.1 (38.8–42.4)

Females					
Variable	time	E (n = 30)	C (n = 24)	E (n = 46)	C (n = 52)
Fat [%]	Pre	26.3 ± 6.6 (24.3–28.2)	26.1 ± 5.3 (24.6–27.5)	24.8 ± 5.9 (22.6–27)	25.4 ± 6.1 (22.8–28)
	Post	24.7 ± 5.2 (23.2–26.3)	26.8 ± 5.6 (25.3–28.4)	24.2 ± 4.9 (22.4–26)	26.3 ± 4.9 (24.3–28.4)
	FU	24.8 ± 6 (23.1–26.6)	25.8 ± 5.9 (24.1–27.4)	24.6 ± 4.9 (22.8–26.5)	26 ± 4.9 (24–28.1)
SBP [mm/Hg]	Pre	121.8 ± 8.4 (119.3–124.3)	120.2 ± 8.8 (117.7–122.7)	120 ± 6.2 (117.7–122.3)	115.5 ± 5.1 (113.4–117.7)
	Post	118.8 ± 6.1 (117–120.6)	119.9 ± 9 (117.4–122.4)	116 ± 6 (113.7–118.3)	115.9 ± 6.4 (113.2–118.6)
	FU	119.2 ± 6.4 (117.3–121.1)	119.7 ± 9.2 (117.1–122.3)	116.2 ± 7.7 (113.3–119)	116.9 ± 6.9 (114–119.8)
DBP [mm/Hg]	Pre	79.6 ± 7.3 (77.4–81.8)	78 ± 7.2 (75.9–80)	77.8 ± 6.2 (75.5–80.1)	75.5 ± 7.6 (72.3–78.7)
	Post	77.6 ± 5.7 (75.9–79.3)	78.3 ± 6.9 (76.4–80.3)	76.4 ± 4.9 (74.6–78.3)	75.6 ± 7.2 (72.6–78.7)
	FU	79.1 ± 6.3 (77.2–80.9)	77.5 ± 7.1 (75.6–79.5)	77.3 ± 4.8 (75.5–79.1)	76.4 ± 7.4 (73.3–79.5)
VO_2max_ [mL/kg/min]	Pre	32.5 ± 5.4 (30.9–34.1)	31.2 ± 6 (29.5–32.8)	32.7 ± 4.3 (31.1–34.3)	33 ± 5.9 (30.5–35.5)
	Post	33.2 ± 5.1 (31.6–34.7)	31.5 ± 6.2 (29.7–33.2)	33.1 ± 4.6 (31.4–34.8)	32.5 ± 6.2 (29.9–35.1)
	FU	30.8 ± 5.5 (29.1–32.4)	31.5 ± 6.4 (29.7–33.3)	30.2 ± 4.8 (28.4–32.1)	32.6 ± 6.6 (29.8–35.4)

### 3.3 Body fat percentage

ANOVA of the body fat percentage showed sexual dimorphism toward more fat tissues in female than male participants (*F* = 268.128, *p* < 0.001, η^2^_pG_ = 0.462, d = 1.31) and effects of the intervention independent of sex (*F* = 3.911, *p* = 0.023, η^2^_pG_ = 0.001, d = 0.03) (Figure 3). However, changes over time were modified by the variant of HIIT used (*F* = 9.900, *p* < 0.001, η^2^_pG_ = 0.001, d = 0.04). Detailed comparisons with post hoc tests revealed no significant differences in body fat percentage (all *p* > 0.05) at baseline. Over the study duration, men in both variant groups showed decreased body fat percentages; however, only the HIIT variant was statistically significant (*p* < 0.001). In the men of both variant groups, the follow-up measurements were similar to the post-intervention measurements, indicating sustained effects that were confirmed by similar fat levels and no statistically significant differences (HIIT variant: *p* = 1.000; HIPT: *p* = 0.075). The female groups also showed decreased body fat percentages, but the effects were reversed between the variants. Female participants practicing HIPT showed a significant difference (*p* < 0.001), while those in the HIIT variant did not (*p* = 1.000). The results were sustained, and the follow-up measurements did not differ significantly from the previous values (both variants: *p* = 1.000). The effects of the interventions on the experimental groups (both men and women) were not large enough to show significant differences from the control groups (all comparisons, *p* = 1.000). The men had significantly lower body fat percentages than the women in each comparison (pre, post, and follow-up) (all *p* < 0.001).

### 3.4 Systolic blood pressure

ANOVA of the SBP showed sex-based differences and higher values in men than women (*F* = 35.404, *p* < 0.001, η^2^_pG_ = 0.095, d = 0.46). Further, the variant of the HIIT showed differences between the groups (*F* = 8.939, *p* = 0.003, η^2^_pG_ = 0.026, d = 0.23). Intervention expressed over time was another factor affecting the results (*F* = 31.801, *p* < 0.001, η^2^_pG_ = 0.012, d = 0.11) (Figure 4). However, the third-order interaction term (time × sex × variant × group: *F* = 2.20, *p* = 0.027, η^2^_pG_ = 0.057, d = 0.25) confirmed that changes over time were related to all of the factors. Detailed comparisons with post hoc tests revealed no significant differences in the SBP (all *p* > 0.05) at baseline. Over the study duration, men in both variant groups showed significantly decreased SBP values (*p* < 0.001 and *p* = 0.016). In the men of both variant groups, the effects were sustained (*p* = 1.000). The female groups also showed decreased SBP values (*p* < 0.001 for both variants) with sustained effects (*p* = 1.000). The effects of the interventions on the experimental groups (both men and women) were not large enough to show significant differences from the control groups (all comparisons, *p* = 1.000). There were no significant differences in SBP values between men and women (experimental and control groups of both variants) (all *p* > 0.100).

### 3.5 Diastolic blood pressure

ANOVA of the DBP showed significant effects of the interventions (time) (*F* = 3.470, *p* = 0.033, η^2^_pG_ = 0.002, d = 0.05). However, these were related to the variant of the HIIT used (*F* = 4.982, *p* = 0.026, η^2^_pG_ = 0.014, d = 0.12) (Figure 5). The second-order interaction term (time × group: *F* = 4.120, *p* = 0.018, η^2^_pG_ = 0.002, d = 0.05) showed that the temporal changes were linked with the experimental groups. Detailed comparisons with post hoc tests revealed no significant differences in the DBP (all *p* > 0.05) at baseline. Over the study duration, despite the decreasing trend of the DBP values, there were no significant differences in the pre–post and follow-up results (both men and women; all *p* = 1.000). Consequently, there were no differences with the control groups (all *p* = 1.000). Moreover, there were no significant differences between the male and female groups (all *p* = 1.000).

### 3.6 Cardiorespiratory fitness (VO_2max_)

ANOVA of the CRF based on VO_2max_ showed a significant effect of the intervention (time) (*F* = 29.390, *p* < 0.001, η^2^_pG_ = 0.014, d = 0.12); however, this was related to sex (*F* = 159.578, *p* < 0.001, η^2^_pG_ = 0.313, d = 0.67) (Figure 6). The second-order interaction term (time × sex × group: *F* = 6.960, *p* = 0.001, η^2^_pG_ = 0.003, d = 0.06) showed that the temporal changes were linked with sex and the experimental groups but not to the variant of HIIT used. Detailed comparisons with post hoc tests revealed no significant differences in CRF between the experimental and control groups (all *p* > 0.05) at baseline. Over the study duration, an increasing trend was observed for the CRF; however, significant improvements were observed in the HIIT group and especially men (*p* < 0.001), while all other differences in the pre–post comparisons were not significant (*p* > 0.05). Follow-up results showed sustained effects and no significant differences (except for women in the HIPT group: *p* = 0.032). There were no significant differences compared to the control groups (all *p* = 1.000). However, all sex-based differences were statistically significant (all *p* < 0.001) in the post-intervention and follow-up measurements.

The results of the DA are limited to the set of variables that have the most deviations between the two groups of participants in terms of the two variants of HIIT. Table 4 presents the calculated statistics for the men and women separately. Only the model built for men was statistically significant (Wilks’ Ʌ = 0.82, *F* = 3.43, *p* = 0.013), while there were no such differences among the women (Wilks’ Ʌ = 0.96, *F* = 0.82, *p* = 0.512). This means that none of the variables could distinguish between the variants in the case of female participants. This was further confirmed through detailed statistical calculations for each of the variables (Table 5). In the case of male participants, two variables were statistically significant, namely, the Δ body fat (*p* = 0.031) and Δ SBP (*p* = 0.033) (Table 5). Both variants of the HIIT differed significantly in terms of the effects on these outcomes. However, these effects were opposition: HIPT supported immense changes in the SBP, whereas HIIT supported changes in BF%.

**TABLE 4:** Descriptive statistics of the delta values for variants and sex.

Variable	HIPT	
	Men (n = 24)	Women (n = 46)
	Mean ± SD (95% CI)	Mean ± SD (95% CI)
ΔFat [%]	−0.67 ± 1.55 (−1.32 to −0.01)	−1.54 ± 2.66 (−2.33 to −0.75)
ΔSBP [mm/Hg]	−5.37 ± 4.32 (−7.2 to −3.55)	−3.07 ± 4.7 (−4.46 to −1.67)
ΔDBP [mm/Hg]	−2.62 ± 6.25 (−5.27–0.02)	−2 ± 5.09 (−3.51 to −0.49)
ΔVO_2max_ [mL/kg/min]	2.14 ± 3.91 (0.49–3.79)	0.67 ± 2.84 (−0.17–1.52)

Variable	HIIT	
	Men (n = 45)	Women (n = 30)
	Mean ± SD (95% CI)	Mean ± SD (95% CI)
ΔFat [%]	−1.31 ± 1.66 (−1.81 to −0.81)	−0.63 ± 2.37 (−1.52–0.25)
ΔSBP [mm/Hg]	−2.73 ± 5.28 (−4.32 to −1.15)	−4 ± 6.89 (−6.57 to −1.43)
ΔDBP [mm/Hg]	−0.22 ± 5 (−1.72–1.28)	−1.37 ± 5.63 (−3.47–0.74)
ΔVO_2max_ [mL/kg/min]	3.81 ± 4.67 (2.41–5.21)	0.41 ± 3.25 (−0.8–1.63)

**TABLE 5:** Wilks’ Lambda (Ʌ), *F*-value, and *p*-value for models built separately for men and women. Partial Wilks’ Lambda (Ʌ_P_), *F*-value, *p*-value, and tolerance are also shown for each variable in each model.

Variable	Wilks’ Ʌ_P_	*F*	*p*	Tolerance
Men: Wilks’ Ʌ = 0.82, *F* = 3.43, *p* = 0.013				
ΔFat [%]	0.93	4.87	0.031	0.87
ΔSBP [mm/Hg]	0.93	4.76	0.033	0.82
ΔDBP [mm/Hg]	0.98	1.49	0.226	0.87
ΔVO_2max_ [mL/kg/min]	0.97	1.80	0.185	0.97
Women: Wilks’ Ʌ = 0.96, *F* = 0.82, *p* = 0.512				
ΔFat [%]	0.97	2.08	0.154	0.99
ΔSBP [mm/Hg]	0.99	0.73	0.396	0.91
ΔDBP [mm/Hg]	0.99	0.54	0.466	0.91
ΔVO_2max_ [mL/kg/min]	0.99	0.04	0.839	0.99

## 4 Discussion

In this study, we investigated the effectiveness of 8-week interventions of two HIIT protocols on body fat percentage, blood pressure values, and CRF. Moreover, the sustainability of the effects was assessed at 8-week follow-up. The effects of both interventions were observed on each variable in both sexes; however, these effects were rather small compared to the control groups. Variation in the effects between the two variants was observed in men but not in women. In men, HIPT had a more significant impact on the SBP, whereas HIIT influenced BF%. Thus, both programs induced positive adaptations; however, these effects appear to be sex-specific and dependent on the type of intervention. The interventions significantly reduced body fat independent of sex, although the changes were more influenced by the HIIT program. Men in the HIIT group showed significant reductions in body fat percentages, which were sustained at follow-up, whereas women in the HIPT group achieved comparable reductions. Despite the initial assumption of differences in the variant of exercise performed, the observed differences between the programs were not significant and were rather dependent on sex within the effects. The interventions also produced significant reductions in SBP in both sexes across both variants, and these reductions were maintained at follow-up in both experimental groups. However, no significant differences were noted between men and women. Despite a general trend toward lower DBP in both sexes, no significant differences were found in the pre–post or follow-up comparisons, and there were no differences compared to the control groups. This suggests minimal effects of the interventions on the DBP. Significant improvements in VO_2max_ were observed, particularly among men in the HIIT group (*p* < 0.001), indicating a positive impact of HIIT on CRF. This effect persisted at follow-up in the men, while women in the HIPT group showed minor improvements. However, the observed changes were not statistically different from those of the control groups, indicating that the HIIT was effective even though the changes were not large enough to significantly differentiate between the experimental and control groups.

Previous studies have demonstrated that even short but intensive intervention protocols, such as the Tabata protocol, could have considerable positive effects on physical fitness, cardiorespiratory system functions, and body composition in youth and adolescents within the limited time available during PE classes (Martin-Smith et al., 2020; Ekstrom et al., 2017; Domaradzki et al., 2020; Ricci et al., 2022). In our previous study, we reported significant decreases in the mean values of bodyweight and body fat percentage in response to HIIT implemented during PE lessons (Domaradzki et al., 2022). However, these effects were observed predominantly among men, suggesting sex as a factor differentiating the effects of HIIT; this was also confirmed in the present study and is likely attributable to differences in the hormonal profiles and muscle fiber compositions between the sexes during adolescence (Xu et al., 2022). Bogataj et al. (2021) reported positive impacts of HIIT on body composition among obese girls, indicating that women can also benefit from HIIT interventions. Racil et al. (2016) showed that programs combining HIIT with plyometric exercises could improve the lean body mass and jumping performance in obese adolescent girls, highlighting the potential of HIPT interventions for women. However, in the present study, we found notable sex-based differences in body fat reduction based on the variant of protocol used. Men experienced significant reductions in body fat percentage with the HIIT, and these results were sustained during the follow-up period. In contrast, women showed comparable reductions when engaging in HIPT. This differential response may be attributed to sex-specific metabolic pathways associated with hormones and differences in body compositions between men and women (González-Gálvez et al., 2024). Dominic and Kishore (2021) demonstrated the effectiveness of interval effort on body fat reduction, which was confirmed in a meta-analysis by Khodadadi et al. (2023). However, our findings indicate that the type of HIIT protocol used could interact with sex to influence the body composition outcomes. The differential responses may be attributable to physiological and hormonal differences between the sexes during adolescence, affecting the mechanisms by which men and women metabolize fat during high-intensity exercises. Additional factors, such as variations in insulin sensitivity, adipokine profiles, and muscle fiber distribution, may also contribute to the observed differences in body fat reduction between the sexes (Lundsgaard and Kiens, 2014; Gado et al., 2024). Our study showed that the improvements in body fat percentage were associated with better CRF—a relationship that is more pronounced in men. This finding aligns with the results reported by Lan et al. (2022) and Guo et al. (2023), who noted associations between fat loss and increased CRF. The utilization of fatty acids during aerobic metabolism may explain the link between reduced body fat and enhanced CRF (Hargreaves and Spriet, 2020). These findings highlight the importance of tailoring the intervention programs to optimize fat reduction and fitness improvements based on sex. Nonetheless, the lack of straightforward differences between the protocols indicates that multiple physiological factors interact to evoke positive adaptations beyond the type of interval training employed (Yu, 2025; Takahashi et al., 2025).

Excess body fat is closely linked to elevated blood pressure, which contributes to hypertension in overweight and obese individuals (Shariq and McKenzie, 2020). Although cardiovascular diseases mainly manifest in the mid-life years, they have their origins in adolescence, making early interventions a public health priority (Chung et al., 2015). Obesity causes functional and structural changes in microcirculation that could impair the microvascular functions underlying elevated blood pressures (El Meouchy et al., 2022). HIIT has been shown to improve cardiovascular health by reducing endothelial damage that precedes atherosclerosis (Tjønna et al., 2009). Tjønna et al. (2009) observed similar improvements in blood pressure independent of sex. Significant reductions in the SBP were observed for both sexes across both intervention variants, and these reductions were maintained during the follow-up period. Delgado-Floody et al. (2019) reported SBP decreases (Δ−8.70 mmHg) in adolescents following HIIT but did not note any relationships between the improvements in CRF and blood pressure. Martínez-Vizcaíno et al. (2014) observed reduced body fat and cardiometabolic risk through improvements in the blood lipid profiles in girls, supporting the effectiveness of HIIT in enhancing the cardiovascular parameters among women. Despite the general trend toward lower DBP, no significant differences were found in our study’s pre–post or follow-up comparisons for either sex, suggesting minimal effects of the interventions on the DBP. This may indicate that the SBP is more responsive to HIIT interventions during adolescence and that sex-specific physiological factors could influence these outcomes. The differential responses between the sexes could be attributed to hormonal variations, vascular adaptations, and differences in autonomic regulation of blood pressure.

CRF is a key indicator of health and a predictor of the risk of cardiovascular diseases (Raghuveer et al., 2020; Belanger et al., 2022). HIIT workouts require less time commitment and have been shown to improve CRF (Carson et al., 2014; Khalafi et al., 2022; Robinson et al., 2015). Our previous study showed improvements in aerobic capacity following HIIT implemented during PE lessons (Domaradzki et al., 2020), with sex-based differences influencing the outcomes. In the present study, significant improvements in VO_2max_ were observed among the men participating in the HIIT group (*p* < 0.001). This positive impact on CRF was sustained during the follow-up period, especially in men. In contrast, women engaging in HIPT showed minor improvements that were not significantly different from those of the control groups. These findings suggest that men may possess a greater capacity for cardiovascular adaptation to high-intensity stimuli, possibly owing to their higher baseline cardiac outputs and muscle masses, thereby facilitating more pronounced improvements in oxygen uptake (Mauro et al., 2024; Svane et al., 2024). This suggests that men may respond more favorably to the HIIT variant in terms of CRF enhancement, while women may require different training stimuli to achieve similar benefits. Burgomaster et al. (2008) revealed no changes in VO_2max_ after HIIT in men, while women exhibited improvements (Talanian et al., 2007). Astorino et al. (2012) also observed differences between the sexes in terms of CRF and blood pressure responses. Our results contrast with some of these findings, showing more significant improvements in boys than girls for both CRF and body fat percentage. Boys may experience greater increases in stroke volume and cardiac output during high-intensity exercises, leading to more substantial improvements in VO_2max_. Hormonal differences, such as higher testosterone levels in men, may enhance their muscle masses and oxygen utilization, contributing to the differential adaptations observed (Pataky et al., 2023). Additionally, fatigue resistance and muscle fiber composition could differ between the sexes, affecting the performance and training outcomes (Pataky et al., 2023). Women may have higher proportions of type I muscle fibers, which are more resistant to fatigue but may not respond as dramatically to high-intensity training as type II fibers that are more prevalent in men (Haizlip et al., 2015). These observations highlight the importance of considering sex-specific responses when designing intervention programs for adolescents. Future research efforts should further investigate the interplay between hormonal, metabolic, and cardiovascular factors to better tailor the HIIT and HIPT protocols for optimizing health outcomes in both sexes. Tailoring the protocols to optimize the benefits for each sex may enhance the effectiveness of the intervention aimed at improving CRF to reduce cardiovascular disease risk. For example, incorporating plyometric exercises may be more beneficial for women, as suggested by the improvements observed in body composition and functional performance in studies like those reported by Racil et al. (2016).

Our research shows that very intensive forms of training with anaerobic metabolism lasting only up to 4 min, when implemented during PE lessons over a period of 8 weeks, can affect body composition and cardiorespiratory system functions. However, the trends in the changes were different depending on the variant of intervention used and sex, indicating the need for further research. These suggest the necessity of studying the dose–response phenomena with intervention time as the load of effort (Domaradzki et al., 2023). At present, it is not clear which variant of the intervention is more efficient and could depend on the measurements and sex. Therefore, future research should investigate hybrid versions of both of the variants considered herein. Future research should also investigate whether adolescents with different weight statuses (e.g., underweight, normal weight, and overweight/obese) respond differently to the interventions. Examining these group-specific effects could provide a more nuanced understanding of how the baseline weights may moderate the impacts of the interventions.

Given the abovementioned findings, we are aware that this study has some limitations that should be addressed in the future. One of the main drawbacks is the lack of control over participants’ caloric intakes and daily physical activities, which are factors that could have influenced the observed outcomes. Additionally, the absence of an assessment of the maturation status of each participant constitutes a significant confounding variable, as variations in the developmental stage could affect the observed results. These limitations should be taken into account when interpreting the findings and also highlight the need for more controlled methodologies in future research.

## 5 Conclusion

Our results prove the positive effects of HIIT exercises lasting only up to 4 min on body fat percentage, blood pressure, and CRF, when implemented during PE lessons over a period of 8 weeks. However, the trends of the changes were different depending on the variant of intervention used and sex of the participant. Body fat reduction was observed in men performing HIIT, while HIPT was observed to be more effective in women. Unlike DBP, SBP showed reductions in both variants of the intervention in both sexes. The effects on CRF were clearer in men performing HIIT than HIPT; however, this was not noted in women irrespective of the variant of intervention performed. The overall effects on the study participants were generally poor, which may be attributed to the short duration of exercises (up to 4 min). Nonetheless, both programs are valuable for PE teachers and practitioners in reducing body fat and blood pressure while enhancing CRF. These interventions can be considered low-dose but feasible owing to their brief duration (4 min) and impact on youth health, making them easy to incorporate into PE lessons. Dedicating such a short portion of the class time can yield positive effects without compromising on the abilities of the teachers to meet their curriculum requirements.

## Data Availability

The raw data supporting the conclusions of this article will be made available by the authors without undue reservation.
